# Barriers to Exercise Participation in Individuals with Fibromyalgia in a Workplace Setting

**DOI:** 10.3390/medicina62020354

**Published:** 2026-02-10

**Authors:** Koulla Parpa

**Affiliations:** Faculty of Sport and Exercise Science, UCLan University of Cyprus, Pyla 7080, Cyprus; kparpa@uclan.ac.uk

**Keywords:** exercise adherence, fatigue, sleep, occupational health, health promotion, chronic pain

## Abstract

*Background and Objectives*: Regular exercise improves pain, fatigue, and overall function in individuals with fibromyalgia (FM), yet adherence remains low, especially among employed adults. This study examined symptom and workplace-related factors associated with exercise participation among employees with FM. *Materials and Methods*: A cross-sectional workplace survey was conducted across nine large employers (>100 employees) representing diverse occupational roles. Participants (*n* = 1044) reported FM diagnosis, exercise participation (≥3 sessions/week), perceived exercise barriers, sleep duration, and job-related stress. Comparisons were conducted between employees with and without FM, and within-group analyses explored exercise-related patterns among those with FM. *Results:* Forty-two participants (4.0%) reported a formal FM diagnosis. Compared with employees without FM, those with FM were older and reported significantly greater pain, fatigue, emotional stress and poorer sleep (all *p* < 0.01). Despite this increased symptom burden, rates of regular exercise did not differ between FM and non-FM employees (40.5% vs. 36.8%, *p* = 0.38). Within the FM group, exercisers and non-exercisers showed minimal observable differences in symptom severity. However, employees with FM reported shorter exercise session durations and identified sleep disruption, fatigue, and work-related demands as prominent barriers. *Conclusions*: Among employed adults with FM, symptom severity alone did not appear to distinguish those who engaged in regular exercise from those who did not. Instead, modifiable workplace and environmental barriers were indicated as key factors influencing exercise participation, emphasizing the need for occupationally informed strategies to support sustained physical activity in this population.

## 1. Introduction

Fibromyalgia (FM) is a chronic pain disorder characterized by widespread musculoskeletal pain, fatigue, as well as sleep and cognitive difficulties that impair daily functioning and quality of life [1,2,3,4,5]. The prevalence of FM in the general population is estimated to range from 1.2% to 5.7%, depending on the classification criteria applied [6]. Despite its relatively high prevalence and disabling symptomatology, the pathophysiology of FM is multifactorial, involving central nervous system sensitization, neuroendocrine and immune dysregulation, peripheral small fiber pathology, genetic susceptibility, and environmental and psychological influences [7].

Exercise is widely recognized as one of the most effective non-pharmacological treatments for FM, leading to significant improvements in both physical and psychological symptoms [8]. Albuquerque et al. [8] reported that regular exercise reduces pain intensity, fatigue, and disease impact scores while enhancing muscle strength balance and overall physical function in individuals with FM. It also improves mood, reduces anxiety and depression, enhances sleep quality and increases vitality and quality of life. Among exercise types, combined and aerobic protocols yield the greatest overall improvements, especially when training intensity progresses gradually. Thus, structured exercise is an effective, low-cost and non-pharmacological treatment that alleviates the primary symptoms and functional limitations experienced by fibromyalgia patients [8]. However, despite these well-documented benefits, adherence to exercise programs among individuals with FM remains low, with approximately one in five participants dropping out [9]. Exercise dropout is a recognized challenge across many clinical populations; however, evidence suggests that dropout rates are high among FM participants. Specifically, a systematic review [9] indicated that individuals with FM discontinue exercise interventions more frequently than participants with other chronic conditions, often due to symptom flare-ups, fatigue and difficulty maintaining participation over time. This raises the question of whether reduced exercise engagement in FM reflects a general adherence issue or a condition-specific barrier that requires targeted investigation. Low-intensity exercise and supervised programs are associated with better adherence, whereas higher body mass index and greater illness impact predict higher dropout rates [9]. This gap between the proven therapeutic value of exercise and real-world adherence emphasizes the need to identify and address personal, environmental and psychological barriers that prevent individuals with FM from engaging in sustained physical activity.

Workplace settings may uniquely amplify these barriers [10]. Occupational demands, such as physically strenuous tasks, prolonged static postures, limited schedule flexibility, and high emotional workload, have been indicated to exacerbate pain, fatigue and cognitive exhaustion in people with FM, thereby reducing their capacity to engage in physical activity [10,11]. Physically demanding roles have been associated with increased musculoskeletal pain and fatigue, while cognitively demanding and managerial positions were associated with mental exhaustion and limited free time, both of which could constrain exercise participation independently of symptom severity [11,12,13]. While previous research has primarily focused on symptom burden and the effects of exercise in clinical populations with FM, providing valuable evidence of exercise efficacy [12], only a few studies have examined how employed individuals with FM engage in and maintain physical activity within the constraints of the work environment [13]. Therefore, exploring the determinants of exercise participation among working adults with FM is essential, as work-related stress and fatigue may significantly influence exercise adherence and participation in this population. Workplace-based studies are critical for understanding how these factors interact to limit exercise participation and overall well-being.

To better understand how multiple factors influence exercise participation among employed individuals with FM, the present study adopts a socio-ecological and occupational health perspective that considers the interaction between individual symptoms, psychosocial influences and workplace conditions [14,15]. The socio-ecological model proposes that health behaviors are shaped not only by individual characteristics but also by interpersonal, environmental and organizational contexts [14]. Accordingly, exercise participation in FM may be understood as a result of interactions between symptom burden (pain, fatigue and sleep disturbance), psychosocial factors and structural workplace conditions such as workload, schedule flexibility and job demands. Occupational stress models further propose that excessive job demands and limited coping resources can lead to fatigue, reduced motivation and decreased engagement in health-promoting behaviors such as physical activity [16]. Therefore, based on the aforementioned perspectives, this study aimed to identify personal and occupational barriers to regular exercise participation among employees with FM and to compare exercise participation patterns between individuals with and without the condition.

## 2. Materials and Methods

### 2.1. Participants

Companies were eligible for inclusion if they employed more than 100 individuals and were located within the state of Arkansas. Initial contact was made with organizational leadership, and surveys were distributed only within companies that provided institutional agreement to participate. Thus, this cross-sectional study analyzed data from a workplace health survey distributed to nine large companies in Arkansas that agreed to participate in the study. The study aimed to explore the prevalence of fibromyalgia symptoms, job-related stress, perceived barriers and exercise participation among working adults. Conducting the research within occupational settings provided an opportunity to assess how job demands and psychological stress influence the ability of individuals with FM to engage in regular physical activity. Ethical approval for this study was obtained from the Institutional Review Board (#04582). All participants provided informed consent prior to participation, and the study was conducted in accordance with the ethical standards of the Declaration of Helsinki.

A total of 1078 responses were received. After excluding incomplete surveys and those who responded “unsure” regarding FM diagnosis, a final sample included 1044 participants. Of these, 42 responders (4.0%) reported being diagnosed with fibromyalgia by a medical professional. Participants represented a range of occupational categories, including executive, middle management and blue-collar roles. Occupational role was used as an indicator for work environment characteristics to assess the differences in physical demands, cognitive load, and emotional workload across job types.

### 2.2. Measures

For this study, the questionnaire was constructed by taking into account the proposed criteria by the American College of Rheumatology for the classification of fibromyalgia [17], the physical and psychological distress experienced by patients with FM, and the fibromyalgia impact questionnaire [18]. A weekly monitor of subjective symptoms developed by Brown et al. (1999) [19] was incorporated into the survey. The questionnaire was designed to serve the study’s purpose and was sent to experts and medical professionals, who were asked to provide comments. After incorporating these comments, a total of 23 questions were incorporated into the survey. Thereafter, content validity was assessed through expert review by eight specialists in the area of rheumatology, exercise science, and occupational health, who rated each item for clarity and relevance on a four-point scale. Item-level and scale-level content validity indices (CVI) were calculated at 0.78 and 0.90, respectively, which were acceptable thresholds for individual items and the overall scale. Furthermore, a pilot group of employees with and without fibromyalgia also provided feedback on wording and comprehension to ensure face validity. In addition, test–retest reliability was assessed in a subset of participants over a seven-day interval using interclass correlation coefficients (ICC) and Cohen’s Kappa (κ) for categorical items, with ICC > 0.70 and κ > 0.60 considered satisfactory. The questionnaire was purposely designed to obtain responses from both patients diagnosed with FM and the non-FM population.

Participants indicated whether they had received a formal fibromyalgia diagnosis by a medical professional (1 = diagnosed and 2 = non-diagnosed). Exercise participation was defined as exercising three or more times per week (yes/no). Participants also reported the average duration of each session (in minutes) and the primary activity (e.g., aerobic, strength, flexibility, or relaxation). Pain precipitating factors (fatigue, emotional stress, movement or overuse, weather changes, and temperature extremes) were treated as barriers. They were used to calculate a barrier burden score representing the total number of perceived exercise barriers. The total number of factors checked (0–10) reflected perceived barriers to exercise. Sleep duration was recorded as the average number of hours slept per night. Job-related physical and emotional stress were rated on a 5-point Likert scale (1 = very low to 5 = very high).

Among participants with FM, the frequency and severity of 27 common FM symptoms (e.g., multiple tender points, fatigue, morning stiffness, numbness, anxiety, temperature sensitivity) were assessed using two 6-point scales (frequency: 0 = never to 5 = daily, severity: 0 = no pain to 5 = severe pain). Frequency and severity scores were summed across all symptoms to generate a total frequency and severity of symptom score (range 0–270), with higher values indicating greater symptom burden. FM participants also reported tender point pain, defined as the total number of anatomical sites (0–10) where pain was experienced. Demographic data, including age, gender and job classification (executive, middle management, or blue collar), was collected for all participants.

### 2.3. Statistical Analysis

Analyses were conducted using SPSS (Version 28; IBM Corp., Armonk, NY, USA). Descriptive statistics summarized demographic characteristics, symptom patterns, and exercise engagement. Independent-samples *t*-tests were used to compare continuous variables (age, symptom burden, stress levels, and hours of sleep) between the FM and non-FM groups. A chi-square test assessed the association between FM diagnosis and regular exercise participation. All analyses excluded “unsure” responses. In addition, an analysis of only FM participants was conducted using independent-samples *t*-tests and Mann–Whitney U tests to compare exercisers and non-exercisers on continuous variables (age, stress, symptom severity, and pain). In addition to binary exercise participation, exercise behavior was examined using frequency, duration and exercise type. To provide a more comprehensive assessment beyond binary participation, exploratory analyses were conducted examining associations between exercise duration, frequency and type and perceived barriers, including sleep and occupational stress variables, among individuals with FM. Among participants with fibromyalgia, non-parametric group comparisons and Spearman correlation analyses were performed to explore relationships between perceived barriers, sleep, stress and exercise characteristics. Normality was evaluated using the Shapiro–Wilk test to determine whether parametric or non-parametric tests were appropriate. Statistical significance was set at *p* < 0.05.

## 3. Results

**Figure 1:**
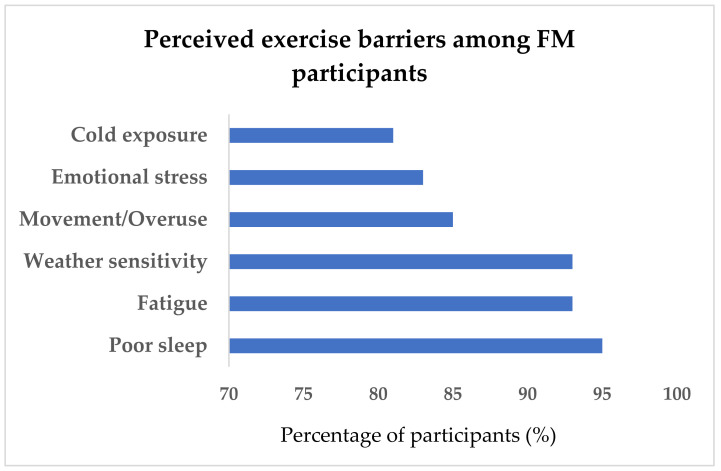
Perceived exercise barriers among FM participants.

After excluding incomplete surveys and those who responded “unsure” regarding FM diagnosis, analyses were conducted on a final sample of 1044 participants. Of these, 42 individuals (4%) reported a formal FM diagnosis by a medical professional, whereas 1002 (96%) reported no diagnosis. Independent samples *t*-tests (Table 1) indicated that participants with FM were significantly older and reported greater emotional stress at work, poorer sleep, and higher pain and symptom scores compared with those without FM. The most frequently reported perceived barriers to exercise participation included poor sleep (95%), fatigue (93%), changing weather (93%), movement or overuse (85%), emotional stress (83%) and cold exposure (81%) (Figure 1).

### 3.1. Gender Distribution

The FM group included six men and 36 women, compared with 291 men and 707 women in the non-FM group. A chi-square test of independence showed a trend toward higher FM prevalence among women than among men, although the difference was not statistically significant [χ^2^(1, N = 1044) = 3.67, *p* = 0.055].

### 3.2. Exercise Participation

A chi-square test examined the association between FM diagnosis and regular exercise participation (Table 2). Around 40% of participants with FM and 36.8% of those without FM reported engaging in regular exercise. This difference did not reach statistical significance [χ^2^(1, N = 1044) = 1.93, *p* = 0.38], suggesting similar exercise participation rates in both groups. Among regular exercisers, individuals with FM reported slightly shorter exercise sessions (37.94 ± 10.62) than non-FM exercisers (43.48 ± 14.29) [t(384) = −2.07, *p* = 0.053], representing a small-to-moderate effect (Cohen’s d = 0.42), despite the difference not reaching statistical significance (*p* = 0.053). Furthermore, among participants with fibromyalgia, perceived barrier burden did not differ significantly between exercisers (6.88 ± 1.96) and non-exercisers (6.56 ± 1.92), with small effect sizes observed. However, exploratory analyses among FM participants who exercised regularly indicated that greater perceived barrier burden and higher physical stress were associated with shorter durations of exercise sessions (Spearman ρ = 0.33 and ρ = −0.35, respectively), although these associations were not statistically significant. Emotional stress showed a weak, non-statistically significant association with exercise duration (ρ = 0.11). The magnitude and direction of the above associations may suggest that perceived barriers and occupational demands may influence exercise duration among those who remain active. Exercise frequency and type were also examined descriptively among individuals with FM. Most individuals who reported regular exercise engaged in low-impact aerobic exercise or flexibility activities performed two to four times per week.

Because the overall comparison indicated no significant difference in exercise participation between FM and non-FM participants, a secondary analysis was conducted focusing exclusively on participants with FM to explore within-group patterns. Descriptive statistics for key variables of only FM participants are presented in Table 3. Independent-samples *t*-tests and Mann–Whitney U tests were conducted to compare exercise and non-exercise groups on age, stress, and pain variables. No statistically significant differences were observed between groups in age, physical stress, emotional stress, total symptom frequency and severity or tender point pain. Effect sizes were small (Cohen’s d = −0.02 to 0.26), suggesting minimal differences between FM exercisers and non-exercisers on these clinical and psychological characteristics. These findings indicate that exercise engagement among individuals with FM may depend on factors beyond demographic or symptom-based characteristics, potentially including motivation, social support or treatment guidance. Given that demographic and symptom factors did not account for exercise engagement within FM participants, occupational influences were next explored.

**Figure 2:**
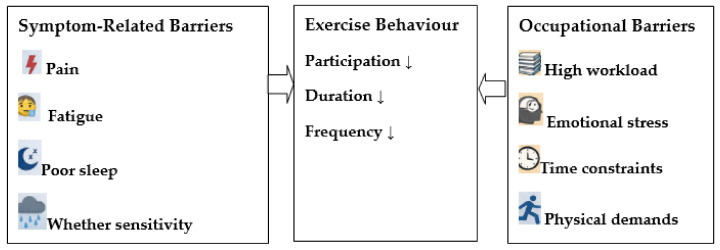
Key symptom-related and occupational factors influencing exercise participation among employees with FM.

To further clarify the relationships between symptom-related and occupational factors influencing exercise behavior among employees with FM, a summary of the key patterns observed in the present study is presented in Figure 2.

Determinants of Exercise Participation in Employees with Fibromyalgia.

### 3.3. Occupational and Functional Limitations

Among participants with FM, 51% reported limiting their work hours due to FM, 29.7% reported being unable to sit or stand for prolonged periods, 18.9% reported being unable to perform heavy physical or mental work, and 82.9% reported limitations in household activities. When analyzed by job category, blue-collar employees reported higher physical stress and fatigue, whereas executive and management staff reported greater emotional stress and time constraints for exercise. Despite differing stressors, both groups identified workplace-related fatigue and workload as major deterrents to consistent exercise participation. These findings highlight that workplace fatigue, time constraints, and role demands may reinforce exercise barriers among employees with FM.

## 4. Discussion

This study identified a small but clinically meaningful subgroup of employees with a confirmed FM diagnosis (4% of the total sample) who reported significantly higher pain and symptom burden, poorer sleep, and greater emotional stress at work than their non-FM peers. Although exercise participation did not differ significantly between the FM and non-FM participants, FM exercisers tended to exercise for shorter durations than their non-FM counterparts. This pattern suggests a potential but modest effect that may not have reached significance due to the small sample size. Within the FM group, exercisers and non-exercisers did not differ in age, stress, sleep duration or tender point pain. These findings indicate that symptom intensity alone does not appear to account for exercise engagement in employed adults with FM. Conclusively, the observed pattern of similar exercise participation despite high symptom burden may help explain the high exercise dropout rates reported in fibromyalgia, indicating that workplace and environmental constraints make it difficult to maintain regular exercise over time.

A key novel contribution of the present study is the finding that symptom severity alone did not distinguish employees with FM who engage in regular exercise from those who do not in this sample. Despite greater pain, fatigue and sleep disturbances, individuals with FM were not less likely to engage in regular exercise than non-FM adults, and within the FM group, exercisers and non-exercisers showed minimal differences in clinical burden. This finding indicates that symptom reduction alone may not necessarily result in increased physical activity and emphasizes the importance of work-related and environmental changes that need to be implemented in order to change the exercise behavior of these individuals. Concurrently, the present study demonstrated that among employees with FM who remained active, greater perceived barriers and higher occupational stress were associated with shorter exercise duration and less consistent participation. Although these associations were exploratory and not statistically significant, they suggest that workplace demands and persistent fatigue may influence not only whether individuals exercise but also the extent to which they are able to sustain regular activity over time.

Participants with FM perceived several interrelated barriers that limited their ability to exercise regularly, most notably poor sleep, chronic fatigue, emotional stress and sensitivity to environmental triggers such as cold temperature and weather changes. These barriers, along with a lack of understanding from others, have been reported to complicate adherence to structured routines further and limit the ability to engage in regular physical activity and exercise [20]. Similarly, a narrative review [21] indicated that climatic and seasonal variations have a significant impact on FM symptoms. Specifically, cold temperatures increase stiffness and pain, and temperature changes can intensify pain and stress, indicating environmental sensitivity [21]. These findings reflect the interaction between symptom variability and self-regulatory fatigue [22] in which ongoing physical stress, discomfort, and environmental sensitivity diminish both the capability and perceived opportunity to engage in regular exercise. Interventions that overlook these day-to-day barriers risk overestimating individuals’ ability to exercise regularly.

The workplace has been demonstrated to be an essential determinant. Both blue-collar and managerial employees cited work-related fatigue and time constraints as obstacles to regular activity. Physical demands predominated in manual roles, while cognitive load and limited schedule flexibility characterized managerial positions. Furthermore, half of the FM participants reported reducing work hours due to their condition, around 30% reported being unable to sit or stand for prolonged hours and 83% experienced restrictions in performing household tasks. These findings are consistent with prior studies showing that work-related fatigue, time pressure and organizational demands significantly limit functional capacity and activity engagement among individuals with FM, often necessitating task adjustments or schedule modifications to maintain employment [11,13,23]. Similarly, occupational characteristics have been linked to FM-related disability, indicating that the nature of work (manual versus managerial) affects both symptom impact and participation [10]. In addition, evidence demonstrates that work context and schedule structure shape physical activity behaviors, with employed women with FM showing higher activity levels than those confined to domestic roles, indicating the role of environmental opportunity rather than symptom severity [13]. Finally, large multicenter surveys have demonstrated that FM frequently reduces work hours and impairs household functioning, indicating that occupational stress influences daily activity [10,14]. These findings demonstrate that structural factors, such as work schedules and time pressure, may limit opportunities for physical activity more than pain itself. When combined with poor sleep and persistent fatigue, these constraints can further diminish motivation and capacity to engage in regular exercise.

Barriers to exercise identified in the present study are not unique to fibromyalgia and have been widely reported across patient populations living with chronic pain and musculoskeletal disorders [24,25] as well as patients with chronic diseases and older adults [26]. Qualitative research has shown that adults with chronic pain perceive multiple obstacles to physical activity, including pain, fatigue, fear of symptom exacerbation, competing life demands, reduced self-confidence and difficulty accessing resources [24]. Similarly, research in individuals with chronic musculoskeletal disorders demonstrated low physical activity levels and frequent reports of fatigue, pain and lack of motivation as major barriers [25]. Finally, an umbrella review of adherence in chronic disease and older populations identified a range of key factors that support continued exercise, including individualization of the exercise program, supervision, education about risks and benefits, social support, communication and feedback, and integration into daily life [26], which may also be applicable to individuals with fibromyalgia.

Notably, these findings suggest that effective interventions such as micro-breaks, flexible scheduling and onsite or near-site low-impact exercise options may create realistic opportunities for exercise engagement. Integrating exercise into work routines could mitigate pain, improve sleep quality and reduce overall symptom load over time. Research indicates that programs such as Zumba [27] and Pilates [28] can improve mood, muscle strength, dynamic balance and quality of life in women, suggesting that more engaging and social exercise formats may promote sustained participation. Furthermore, immersive virtual reality-based exercise interventions have shown promising effects on pain management and engagement in individuals with FM, potentially enhancing both adherence and outcomes [29]. In addition, progressive resistance exercise may provide substantial benefits in reducing pain, enhancing functionality and improving overall quality of life [30] when implemented at gradually increasing intensities tailored to individual capacity, along with appropriate rest periods to ensure safety. Also, recent evidence indicates that aerobic training combined with blood flow restriction is a feasible and safe innovation that can enhance cardiorespiratory fitness, strength and balance, while reducing stiffness and symptom impact in women with FM [31]. This exercise approach may represent a novel, time-efficient alternative for individuals seeking exercise modalities tailored to their physical and time constraints.

These findings emphasize that improving exercise participation among individuals with FM requires not only personal and workplace-level interventions but also systemic improvements in healthcare. Recent work from Atlantic Canada [32] demonstrated that integrating Clinical Exercise Physiologists into primary care teams, supported by more precise referral mechanisms and regulatory frameworks, could help bridge existing gaps in exercise promotion and management. Expanding such models across occupational and primary care settings could strengthen multidisciplinary efforts to sustain physical activity among people with FM.

### Limitations

In addition to the study’s cross-sectional design, all measures were self-reported, which may have introduced recall bias. Furthermore, the small number of FM participants limits the power to detect small within-group effects. In addition, data collection occurred in December, when elevated workload and holiday stress may have amplified fatigue and time constraints.

## 5. Conclusions

Among employed adults with FM, greater symptom burden does not necessarily translate to lower exercise participation. Instead, exercise engagement appears constrained by modifiable workplace and environmental barriers. Addressing these organizational determinants alongside clinical symptom management may be vital for achieving optimal exercise participation among people with fibromyalgia.

## Figures and Tables

**Table 1:** Characteristics by FM diagnosis presented as means ± standard deviation (SD).

Variable	FM (n = 42)	Non-FM (n = 1002)	t	*p*
Age (years)	49.71 ± 7.22	43.39 ± 11.83	5.38	<0.001
Years of work	10.80 ± 8.52	9.65 ± 8.66	0.86	0.390
Physical stress at work (0–5)	3.10 ± 1.27	2.84 ± 0.99	1.30	0.200
Emotional stress at work (0–5)	3.98 ± 0.81	3.57 ± 1.00	3.14	0.003
Total frequency and severity of symptoms (0–270)	139.8 ± 41.07	46.40 ± 20.08	14.66	<0.001
Total tender point pain (0–10)	7.02 ± 1.87	1.39 ± 1.67	19.24	<0.001
Hours of sleep	5.31 ± 1.24	6.77 ± 1.09	−7.49	<0.001

Note: FM: individuals with fibromyalgia.

**Table 2:** Exercise Participation by FM diagnosis.

FM Diagnosis	Exercisers (*n*)	Non-Exercisers (*n*)	% Exercising
FM	17	25	40.5
Non-FM	369	633	36.8

Note: FM: fibromyalgia individuals.

**Table 3:** Participant characteristics by exercise status (FM participants only).

Variable	Test Used	Exercisers	Non-Exercisers	*p*
Age (years)	*t*-test	50.82 ± 7.59	48.96 ± 7.01	0.43
Physical stress at work (0–5)	Mann–Whitney U	3.00 ± 1.06	3.16 ± 1.40	0.64
Emotional stress at work (0–5)	Mann–Whitney U	3.94 ± 0.66	4.00 ± 0.91	0.62
Total frequency and severity of symptoms (0–270)	*t*-test	136.7 ± 37.30	141.9 ± 44.07	0.68
Total tender point pain (0–10)	Mann–Whitney U	7.00 ± 1.80	7.04 ± 1.95	0.90

Note: FM: fibromyalgia individuals.

## Data Availability

The raw data supporting the conclusions of this article will be made available by the author upon request.

## References

[1] Garcia Rodriguez D.F., Abud Mendoza C. (2020). Physiopathology of fibromyalgia. Reumatol. Clin..

[2] Jurado-Priego L.N., Cueto-Ureña C., Ramírez-Expósito M.J., Martínez-Martos J.M. (2024). Fibromyalgia: A review of the pathophysiological mechanisms and multidisciplinary treatment strategies. Biomedicines.

[3] Flynn D. (2023). Chronic pain syndromes: Fibromyalgia. FP Essent..

[4] Zheng C., Zhou T. (2022). Effect of acupuncture on pain, fatigue, sleep, physical function, stiffness, well-being, and safety in fibromyalgia: A systematic review and meta-analysis. J. Pain Res..

[5] Bertolucci P.H., de Oliveira F.F. (2013). Cognitive impairment in fibromyalgia. Curr. Pain Headache Rep..

[6] Jones G.T., Atzeni F., Beasley M., Flüß E., Sarzi-Puttini P., Macfarlane G.J. (2015). The prevalence of fibromyalgia in the general population: A comparison of the American College of Rheumatology 1990, 2010, and modified 2010 classification criteria. Arthritis Rheumatol..

[7] Gyorfi M., Rupp A., Abd-Elsayed A. (2022). Fibromyalgia pathophysiology. Biomedicines.

[8] Albuquerque M.L., Monteiro D., Marinho D.A., Vilarino G.T., Andrade A., Neiva H.P. (2022). Effects of different protocols of physical exercise on fibromyalgia syndrome treatment: Systematic review and meta-analysis of randomized controlled trials. Rheumatol. Int..

[9] Vancampfort D., Van Damme T., Brunner E., McGrath R.L., Hemmings L., Guimaraes M.E., Schuch F. (2024). Dropout from exercise interventions in adults with fibromyalgia: A systematic review and meta-analysis. Arch. Phys. Med. Rehabil..

[10] Fitzcharles M.A., Ste-Marie P.A., Rampakakis E., Sampalis J.S., Shir Y. (2016). Disability in fibromyalgia associates with symptom severity and occupation characteristics. J. Rheumatol..

[11] Rakovski C., Zettel-Watson L., Rutledge D. (2012). Association of employment and working conditions with physical and mental health symptoms for people with fibromyalgia. Disabil. Rehabil..

[12] Wang C., Schmid C.H., Fielding R.A., Harvey W.F., Reid K.F., Price L.L., Driban J.B., Kalish R., Rones R., McAlindon T. (2018). Effect of tai chi versus aerobic exercise for fibromyalgia: Comparative effectiveness randomized controlled trial. BMJ.

[13] Girela-Rejón M.J., Gavilán-Carrera B., Aparicio-Ortega E., Borges-Cosic M., García-Rodríguez I.C., Delgado-Fernández M., Estévez-López F. (2020). Is type of work associated with physical activity and sedentary behaviour in women with fibromyalgia? A cross-sectional study from the al-Ándalus project. BMJ Open.

[14] Golden S.D., Earp J.A. (2012). Social ecological approaches to individuals and their contexts: Twenty years of health education & behavior health promotion interventions. Health Educ. Behav..

[15] Uchendu C., Windle R., Blake H. (2020). Perceived Facilitators and Barriers to Nigerian Nurses’ Engagement in Health Promoting Behaviors: A Socio-Ecological Model Approach. Int. J. Environ. Res. Public Health.

[16] Davidson M.J., Cooper C.L. (1981). A model of occupational stress. J. Occup. Environ. Med..

[17] Wolfe F., Smythe H.A., Yunus M.B., Bennett R.M., Bombardier C., Goldenberg D.L., Tugwell P., Campbell S.M., Abeles M., Clark P. (1990). The American College of Rheumatology 1990 criteria for the classification of fibromyalgia. Arthritis Rheum..

[18] Burckhardt C.S., Clark S.R., Bennett R.M. (1991). The fibromyalgia impact questionnaire: Development and validation. J. Rheumatol..

[19] Brown B. (1999). Weekly monitor of subjective symptoms. ACSM Health Fit. Summit.

[20] Russell D., Álvarez-Gallardo I.C., Wilson I., Hughes C.M., Davison G.W., Sañudo B., McVeigh J.G. (2018). “Exercise to me is a scary word”: Perceptions of fatigue, sleep dysfunction, and exercise in people with fibromyalgia syndrome—A focus group study. Rheumatol. Int..

[21] Bazzichi L., Giorgi V., Di Franco M., Iannuccelli C., Bongiovanni S., Batticciotto A., Pellegrino G., Sarzi-Puttini P. (2024). Environmental factors and fibromyalgia syndrome: A narrative review. Clin. Exp. Rheumatol..

[22] Bartley E.J., Robinson M.E., Staud R. (2018). Pain and fatigue variability patterns distinguish subgroups of fibromyalgia patients. J. Pain.

[23] Mannerkorpi K., Gard G. (2012). Hinders for continued work among persons with fibromyalgia. BMC Musculoskelet. Disord..

[24] Vader K., Doulas T., Patel R., Miller J. (2021). Experiences, barriers, and facilitators to participating in physical activity and exercise in adults living with chronic pain: A qualitative study. Disabil. Rehabil..

[25] Kalın A.S., Aytur Y.K. (2023). Physical activity levels of individuals with chronic musculoskeletal disorders: Their relationship with barriers and facilitators. Musculoskelet. Care.

[26] Collado-Mateo D., Lavín-Pérez A.M., Peñacoba C., Del Coso J., Leyton-Román M., Luque-Casado A., Gasque P., Fernandez-del-Olmo M.A., Amado-Alonso D. (2021). Key factors associated with adherence to physical exercise in patients with chronic diseases and older adults: An umbrella review. Int. J. Environ. Res. Public Health.

[27] Ben Waer F., Lahiani M., Alexe C.I., Badau D., Onoi M.P., Alexe D.I., Sahli S. (2024). The effects of Pilates vs. Zumba dancing on functional performance, mood and health-related quality of life in postmenopausal women. Appl. Sci..

[28] Nithuthorn C., Chaipichit N., Jeeraaumponwat T., Maiprasert M., Dilokthornsakul P. (2024). Effect of Pilates on pain and health-related quality of life in fibromyalgia patients: A systematic review and meta-analysis. J. Clin. Med..

[29] Carvajal-Parodi C., Arias-Álvarez G., Ulloa-Díaz D., Romero-Vera L., Andrades-Ramírez O., Guede-Rojas F., Ponce-González J.G. (2025). Immersive virtual reality-based exercise for pain management in fibromyalgia: An exploratory study with risk of poor outcomes stratification. Appl. Sci..

[30] Guachizaca Moreno K.P., Flores-Santy L.F., Vinueza Fernández I. (2025). Intensity of resistance exercise and its effects on pain, functionality, and quality of life in adults with fibromyalgia: A systematic review. J. Funct. Morphol. Kinesiol..

[31] Rodríguez-Bautista J.C., López-Lluch G., Rodríguez-Torres P., López-Moral Á., Quijada-Carrera J., Bueno-Antequera J., Blanco-Suárez M., Cáceres-Calle Ó., Munguia-Izquierdo D. (2024). Feasibility, safety, and effects of an aerobic training program with blood flow restriction on functional capacity and symptomatology in women with fibromyalgia: A pilot study. Biomedicines.

[32] Ryan J.M., Cameron E.M., Button D.C., McGowan E. (2022). Using a socioecological approach to explore the integration of exercise physiologists into primary healthcare teams. Int. J. Sport Exerc. Health Res..

